# Intelligent Human–Computer Interaction: Combined Wrist and Forearm Myoelectric Signals for Handwriting Recognition

**DOI:** 10.3390/bioengineering11050458

**Published:** 2024-05-04

**Authors:** Andrea Tigrini, Simone Ranaldi, Federica Verdini, Rami Mobarak, Mara Scattolini, Silvia Conforto, Maurizio Schmid, Laura Burattini, Ennio Gambi, Sandro Fioretti, Alessandro Mengarelli

**Affiliations:** 1 Department of Information Engineering, Università Politecnica delle Marche, 60131 Ancona, Italy; f.verdini@staff.univpm.it (F.V.); r.mobarak@pm.univpm.it (R.M.); m.scattolini@pm.univpm.it (M.S.); l.burattini@staff.univpm.it (L.B.); e.gambi@staff.univpm.it (E.G.); s.fioretti@staff.univpm.it (S.F.); a.mengarelli@staff.univpm.it (A.M.); 2 Deparment of Industrial, Electronics and Mechanical Engineering, Roma Tre University, 00146 Rome, Italy; simone.ranaldi@uniroma3.it (S.R.); silvia.conforto@uniroma3.it (S.C.); maurizio.schmid@uniroma3.it (M.S.)

**Keywords:** EMG, handwriting, pattern recognition, feature extraction, signal processing, human–machine interface

## Abstract

Recent studies have highlighted the possibility of using surface electromyographic (EMG) signals to develop human–computer interfaces that are also able to recognize complex motor tasks involving the hand as the handwriting of digits. However, the automatic recognition of words from EMG information has not yet been studied. The aim of this study is to investigate the feasibility of using combined forearm and wrist EMG probes for solving the handwriting recognition problem of 30 words with consolidated machine-learning techniques and aggregating state-of-the-art features extracted in the time and frequency domains. Six healthy subjects, three females and three males aged between 25 and 40 years, were recruited for the study. Two tests in pattern recognition were conducted to assess the possibility of classifying fine hand movements through EMG signals. The first test was designed to assess the feasibility of using consolidated myoelectric control technology with shallow machine-learning methods in the field of handwriting detection. The second test was implemented to assess if specific feature extraction schemes can guarantee high performances with limited complexity of the processing pipeline. Among support vector machine, linear discriminant analysis, and K-nearest neighbours (KNN), the last one showed the best classification performances in the 30-word classification problem, with a mean accuracy of 95% and 85% when using all the features and a specific feature set known as *TDAR*, respectively. The obtained results confirmed the validity of using combined wrist and forearm EMG data for intelligent handwriting recognition through pattern recognition approaches in real scenarios.

## 1. Introduction

Nowadays, the role of surface electromyography (EMG) is central in the development of smart assistive technologies. This is certainly due to the large amount of tools available in the field of signal processing and machine learning. Indeed, the literature provides a variety of software packages that facilitate the training and validation of EMG-based human–machine interfaces able to decode the human intent of motion within a given set of movements [1,2]. Such interfaces find application in prosthetic control of upper and lower bionic limbs [3,4], but also for the realization of intelligent human–computer interactions in virtual and augmented reality, and for biometric identification [5,6,7,8]. Myoelectric interfaces can also find application in modern scenarios since they can be used to decode handwritten characters or digits [9,10], supporting the development of immersive rehabilitation protocols with a consistent involvement of the cognitive centres of the brain [11].

Many pieces of evidence in the literature suggest the use of strengthening the handwriting motor skills to mitigate the effects carried by the pathology [12,13,14]. In children affected by dysgraphia, for instance, handwriting exercises delivered through human–machine interfaces can be particularly beneficial as they may provide timely feedback to correct their style [13]. Another important rehabilitation scenario of handwriting involves Parkinson’s disease, where handwriting difficulties occur frequently and are generally known as micrographia, i.e., the reduction of writing amplitude, eventually resulting in a reduced legibility [15]. In this context, past studies have shown that handwriting exercises help improve the writing size in those subjects affected by Parkinson’s disease [16,17]. Another important application of handwriting rehabilitation is related to motor hand ability recovery in patients after a severe traumatic brain injury, or after coma. In both cases, the use of handwriting showed improvements in patient condition, showing progressively improved adherence to recovery of normal functionalities of the hands [18,19,20].

It should be noted that EMG-based interfaces for handwriting recognition software have been less investigated compared to other technologies that generally employ pattern recognition methods trained with 2D images recorded on touch screen tablets, which can also associate pressure information of the pen [21,22]. However, these data sources did not reflect the actual volition of human movement, and handwriting is recognized only after having complete 2D information [22]. On the other hand, EMG can directly mirror the motor control volition and data and can be used in a sequential manner to create architectures that recognize the handwriting online., i.e., with an update frequency that can be used to making the interaction more fluent [10]. Hence, although EMG has been considered as a potential option for handwriting recognition, only a few studies have explored its use in classifying digits or letters [9,10]. The recognition of handwritten characters from EMG data has been tackled using template matching, dynamic time warping, and deep-learning methods [9,23,24]. However, these approaches require large databases and impose a high computational burden during the training phase, making the practical applicability of such architectures limited. Indeed, in [9], each participant wrote 36 characters on a screen, and each character was repeated one thousand times to create a dataset useful to train deep-learning-based models. Confirmation regarding the large amount of data for delivering reliable EMG-based deep-learning models can also be found in the recent literature [8], especially when one would realize plug-and-play devices. Furthermore, similar to [24], the EMG data were first mapped to pen coordinates before the classification step, which was obtained from a tablet. Such aspects may challenge the applicability of EMG-based human–computer interfaces when they need to be tailored on specific subjects [10].

Hence, the development of reliable and fast-calibrating myoelectric interfaces based on shallow pattern recognition technique results are still appealing and deserve to be investigated. In passing, recent studies have reported advancements in the EMG electrode locations for the development of human–computer interfaces able to extend the capability of the human being in smartly interacting with software [8]. To do this, the recent literature has focused on showing the importance of the forearm and wrist as a good location to acquire EMG for the development of transferable architectures in real scenarios [8,10,25,26]. However, the full potentialities of the aforementioned architectures were not completely investigated in complex handwriting tasks as the recognition of a set of words. Hence, the aim of this study is to investigate the feasibility of using combined forearm and wrist EMG signals to develop a 30-word handwriting recognition architecture. For this reason, five consolidated feature sets in the field of myoelectric control were employed to extract signal characteristics in the time and frequency domains. Moreover, the possible advantage of opportunely smoothing the classifier output signal was also assessed by applying a majority voting approach [27].

The paper is thus organized as follows: Section 2 reports the experimental protocol, the methodologies for processing EMG data, the pattern recognition algorithms, and the metrics used for assessing classification performances. For results and discussion, respectively, Section 3 and Section 4 present the performances obtained for all the classifiers with respect to the five feature sets employed and the implications of using such models with and without correction strategies. Finally, Section 5 concludes the manuscript with the main findings of the present work and possible associated limitations.

## 2. Materials and Methods

### 2.1. Study Population

A total of six healthy subjects, three females and three males aged between 25 and 40 years, were recruited for the study considering an age range in accordance with [10,25,28]. Subjects did not manifest any impairment or cognitive disturbance, and they were not affected by neurological disorders. Before applying for the study, each subject was informed about the experimental procedure, which involved the use of commercial devices with conformity declaration approval. All of them provided written consent to participate in the experiment, which was conducted following the protocols of the Declaration of Helsinki.

### 2.2. Experimental Protocol

**Figure 1:**
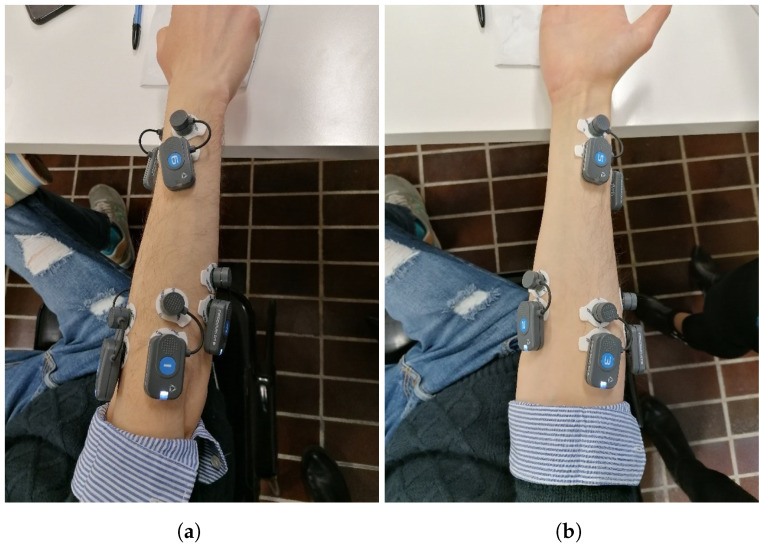
Electromyographic (EMG) probes location in the dorsal (panel (**a**)) and medial (panel (**b**)) view of the arm. This setup was used to record the electrical activity of the wrist and forearm muscle for each subject. All probes was placed by the same expert in order to minimize effects due to inter-operator variability.

All participants were equipped with a total of six surface EMG electrodes with a sampling frequency of 1000 Hz that operated synchronously (FREEEMG system, BTS-Bioengineering, Milan, Italy). The electrodes were placed by the same expert operator over the forearm and wrist in order to reduce the inter-operator variability. More specifically, the first four electrodes were employed for covering the forearm: electrodes 1 and 4 were placed on the extensor digitorum and the flexor carpi ulnaris, whereas electrodes 2 and 3 recorded the myoelectric activity of the flexor carpi radialis and the brachioradialis [10]. The last two electrodes, i.e., electrodes 5 and 6, were placed respectively on the distal ending of the flexor carpi radialis in correspondence of the deep flexor pollicis longus, and in correspondence of the extensor digiti minimi (Figure 1). Such a configuration guaranteed a wide spatial coverage of the arm, facilitating the recording of EMG signals from the bellies of the deep layer of muscles involved in the control of fine finger and wrist movements [29,30]. All the experimental sessions were conducted within the Movement Analysis Laboratory of the Università Politecnica delle Marche.

After being instrumented, all subjects were asked to sit on a chair in a comfortable posture for writing with their dominant hand in their natural cursive style. Then, they were asked to take a pen and write on a sheet of paper a total of 30 words, i.e., respectively, 10 nouns, adjectives, and verbs chosen among the most commonly employed in English (http://oxforddictionaries.com (accessed on 22 of May 2023); see Table 1). Each word was written 10 times with a pause of 5 s between two consecutive written words. The number of repetitions was selected to obtain a consistent amount of data for creating balanced training–validation and testing folds. This mitigated possible biases in the computation of the performance metrics, and at the same time allowed us to keep the acquisition time within reasonable limits [4]. Moreover, after completion of a sequence, each subject was asked to take a break for at least two minutes to prevent fatigue. During each rest period, the EMG data were saved in an appropriate folder and the subject was ready to face another trial. Each trial contained information relative to a specific word, and subjects proceeded by writing nouns, adjectives, and verbs in the order indicated by the class reported in Table 1. Eventually, for each subject a total of 30 recordings, i.e., one per each word containing 6 EMG signals with 10 activation events, were available for pre-processing and feature extraction.

### 2.3. Data Pre-Processing and Feature Extraction

**Figure 2:**
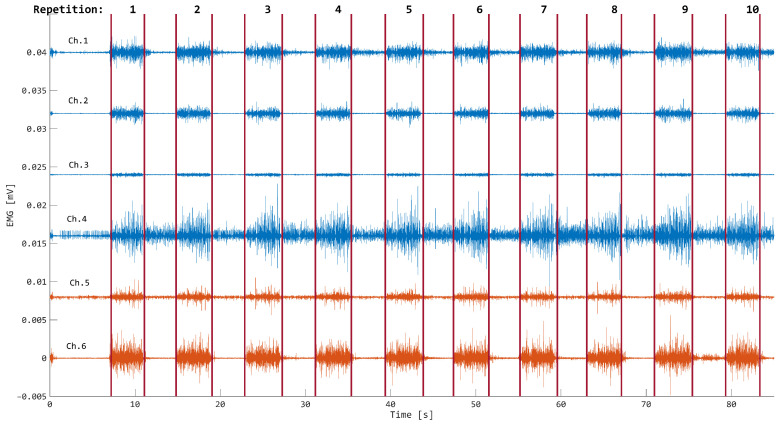
Segmentation of forearm (blue traces) and wrist (orange traces) EMGs performed to identify the handwriting 10-fold repetition of the word “great” by subject 2.

For each record, EMG signals were filtered with a fourth-order, zero-phase Butterworth band-pass filter between 35 Hz and 450 Hz. Then, muscle onset state for data segmentation was opportunely identified with the threshold method [31,32]. An example of the segmentation step for a data record is reported in Figure 2.

The six EMG signals associated with each word repetition were segmented to create a database, allowing for the extraction of features in both the time and frequency domains (TD and FD) [1,4]. A sliding window of 150 ms with an overlap of 75 ms was used to calculate the features listed in Table 2 [10].

For each subject, a matrix was created with a number of rows corresponding to the total number of segmented windows and a number of columns corresponding to all the features computed for each channel. Data at feature level were then aggregated according to the feature sets that are commonly used for myoelectric pattern recognition [1,10]. In this study, five sets of characteristics were considered, the first being the *Hudgins* set, which consists of four time-domain features (MAV, WL, SSC, ZC) [33]. The second set was the *Du* set, which includes six time-domain features i.e., IEMG, VAR, WAMP, WL, SSC, ZC [34]. Two other feature sets proposed in [1] were also taken into account: *Phinyomark 1*, which is composed of five time-domain and two frequency-domain features, i.e., MAV, WL, WAMP, ZC, AR, MNF and PSR, and *Phinyomark 2*, which is made up of four time-domain features, i.e., WPermEn, CC, RMS and WL. Finally, the last feature set considered was *TDAR*, which is composed of seven features, namely MAV, SSC, WL, VAR, WAMP, AR, and ZC [35].

### 2.4. Classification Algorithms

In this study, three state-of-the-art classification algorithms were used for myoelectric control: linear discriminant analysis (LDA), support vector machine (SVM), and K-nearest neighbours (KNN) [1,4,36]. Concerning the LDA, it is a statistical learning model that has demonstrated applicability in myoelectric hand gesture recognition [1,3,37]. Here, a principal diagonal covariance matrix model was assumed to model the data [37]. Regarding the SVM, a linear kernel was employed, and a one-versus-one approach was used to deal with the multi-class nature of the handwriting recognition problem through SVM [37,38]. Finally, KNN is a non-parametric machine-learning model that requires the definition of the number of neighbours (k) to consider, and a distance metric between data points [37,38]. In this case, k was set equal to 2 and Euclidean norm was used.

### 2.5. Pattern Recognition Tests

Two pattern recognition tests were conducted to assess the possibility of classifying fine hand movements, that is writing 30 words, through EMG-based pattern recognition. In the first one, all the features in TD and FD were combined and used to train and test intra-subject models. Thus LDA, SVM, and KNN were trained for each subject using a five-fold cross validation scheme on 70% of the subject’s data, while the remaining 30% was used for testing. As done in previous studies [4,10], attention was paid to balancing the data split in each stage, i.e., learning, validation, and testing, to reduce bias in the models, thus avoiding overoptimistic results.

The second test was conducted to evaluate the classification performance of the aggregated sets reported in Section 2.3. This experiment was implemented to determine if consolidated feature extraction schemes can be useful in a complex classification problem, such as the one faced in this study, which involves more precise control of the wrist and hand movement. To do this, LDA, SVM, and KNN were trained to model intra-subject EMG-based classification of the 30 written words using the aforementioned training–validation and classification scheme. Thus, the *Hudgins*, *Du*, *Phinyomark 1*, *Phinyomark 2*, and *TDAR* sets were split into 70% of training data, which were then used in a five-fold cross validation scheme, while the remaining 30% was used for testing. It is worth noting that, in both experiments, the training and testing sets were split randomly but balanced, as suggested in [10].

Moreover, the effect of smoothing the classification output using majority voting (MV) was investigated, as suggested when classifiers have to be used in real scenarios [29,39]. MV is a post-processing approach used to enhance the model’s performance, and it utilizes a stream of class decisions that resulted from a sliding window with an overlapping scheme to reduce any potential noisy decision [40]. More formally, given a streamed number of votes, *M*, from the classifier, the smoothed MV decision, $d_{\mathrm{MV}}$, can be expressed as

(1) $$d_{\mathrm{MV}}=\arg\max_{c}\sum_{i=-\frac{M}{2}}^{\frac{M}{2}}I\left(d_{i}=c\right),$$

where *c* represent the class label and $I(d_{i}=c)$ is an indicator function that equals 1 if the *i*th vote, $d_{i}$, is equal to class *c*, and 0 otherwise [27,29,39]. The number of votes, *M*, is determined by the processing time, the time consumed during feature extraction and classification, and the acceptable delay, which is the response time of the control system [27]. In this study, *M* was set to 4.

### 2.6. Performance Metrics and Statistical Analysis

To properly assess the results obtained in the pattern recognition tests described in Section 2.5, three metrics have been taken into account during the testing of the models, i.e., the accuracy, the F1 score, and the Matthews correlation coefficient (MCC). The accuracy can be computed as the ratio between the correct predictions over the total predictions made, more formally:

(2) $$\mathrm{Acc}=\frac{TP+TN}{TP+FP+TN+FN},$$

where TP, TN, FP, and FN are, respectively, the number of true positives, true negatives, false positives, and false negatives.

The second metric used to assess the goodness of the performances is the F1 score, which is the harmonic mean between recall and precision. Such a metric combines a measure of the ability to correctly categorise the cases, with an index of robustness, given by the proportion of instances that are not missed. The F1 score determines how well the model performs and it is computed as

(3) $$F1=\frac{\mathrm{Precision}\cdot \mathrm{Recall}}{\mathrm{Precision}+\mathrm{Recall}},$$

where

(4) $$\mathrm{Precision}=\frac{TP}{TP+FP}$$

(5) $$\mathrm{Recall}=\frac{TP}{FN+TP}.$$

The last metric employed was the MCC, which is more sensitive to unbalanced datasets. Thus, although in this study attention was paid to balancing the split between training and testing data, MCC can be employed to confirm or not the indication given by the accuracy metric. The MCC metric can range between −1 and 1, and positive values close to 1 indicate a good performances of the model. Such a metric can be computed as

(6) $$\mathrm{MCC}=\frac{TP\cdot TN-FP\cdot FN}{\sqrt{\left(TP+FP)\cdot (TP+FN)\cdot (TN+FP)\cdot (TN+FN\right)}}.$$

The statistical differences between different conditions were assessed using the Wilcoxon paired rank sum test, with statistical significance set at 0.05.

## 3. Results

Table 3 summarizes the results obtained testing for the KNN, SVM, and LDA models. The KNN showed good classification performances in the 30-word classification problem, with a mean accuracy of 85%. Such goodness is also confirmed by F1 and MCC, which yielded values of 84%. On the other hand, SVM and LDA showed poor performances, with a mean accuracy not superior to 65% and 37%, respectively. These results were confirmed by MCC and F1, which indicated that the use of all the features grouped together, if fed to the LDA and SVM models, did not lead to robust results among the subjects.

**Figure 3:**
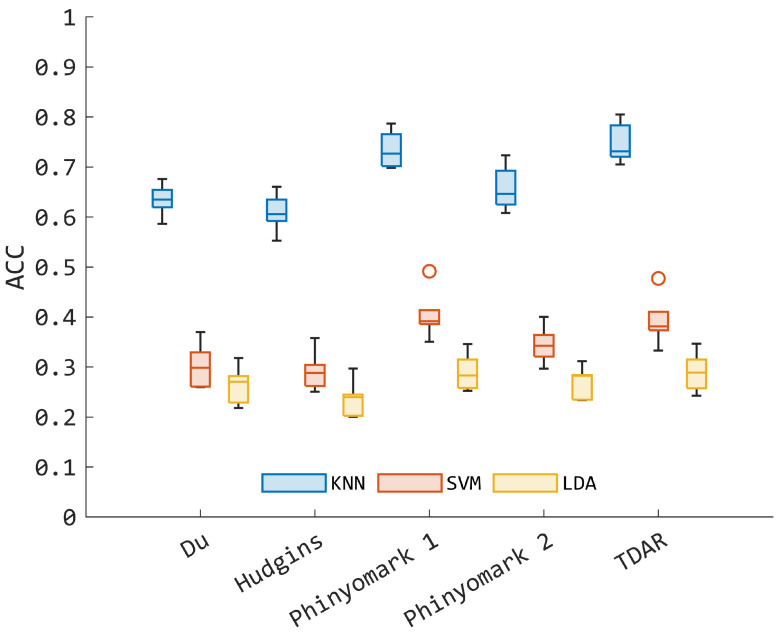
Mean accuracy (ACC) among the subjects obtained with respect to the specific feature sets considered (see Section 2.5), reported in the x-axis of the figure.

**Figure 4:**
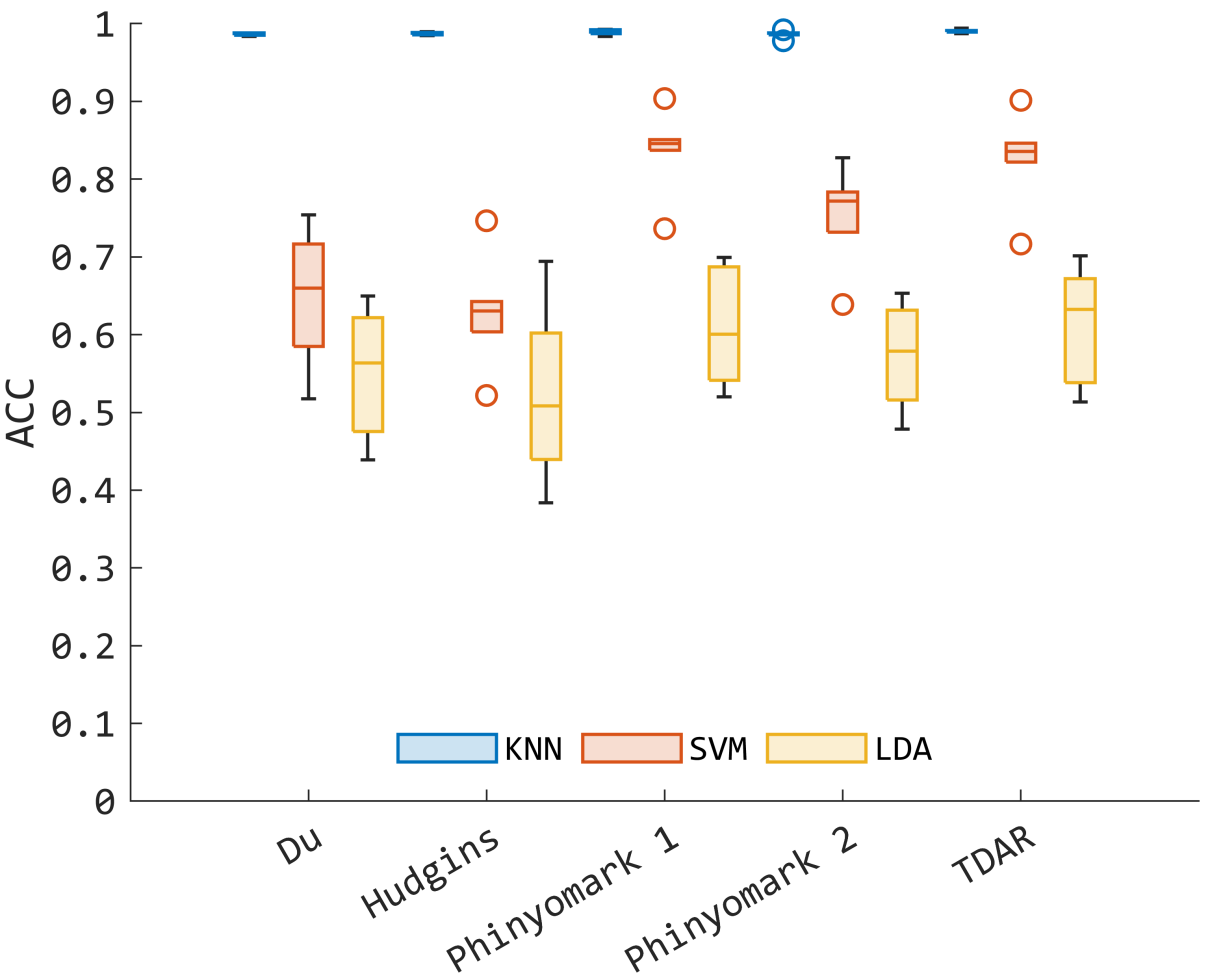
Mean accuracy (ACC) among the subjects obtained with respect to the specific feature sets considered (see Section 2.5), reported in the x-axis of the figure. In this case, the effect of using the majority voting (MV) approach is highlighted with respect to Figure 3.

Regarding the second experiment, Figure 3 summarizes the accuracy obtained in testing for the five sets and for the three machine-learning models used. As confirmed in the first experiment, KNN outperformed both SVM and LDA, with the latter showing the worst performancs. These results are also confirmed when the MV scheme is applied, as reported in Figure 4.

The KNN model had an average accuracy higher than 70%, with the *TDAR* and *Phinyomark 1* sets performing significantly better than the other sets (*p* < 0.05). However, when comparing the two sets, there was no significant difference in the mean accuracy (*p* = 0.5887). The mean accuracy for *TDAR* and *Phinyomark 1* was 74.6% and 73.4%, respectively, with no relevant difference in the interquartile range (see Figure 3). This supports the lack of a significant difference between the two sets. The same can be said for the other two classification algorithms, with the *TDAR* set having the best performance metrics, even with poor performances, i.e., average accuracy of 39.3% for the SVM and of 29.0% for the LDA algorithm without the MV application.

Concerning the application of MV, it should be noted that it boosted the performances of all the classifiers employed. In the case of KNN (see Figure 4), the role of feature sets is completely lost, as no significant differences were found when comparing all five sets. This indicates that the applied post processor consistently improved the classification performance up to a mean accuracy that is higher than 95%. However, if one keeps the focus on the SVM model (Figure 4), *TDAR* and *Phinyomark 1* confirmed their superior performances compared to the other sets (*p* < 0.05), with a non-significant difference between the two. In this case, LDA showed poor performance in terms of accuracy, with large variability among the subjects (Figure 4). Moreover, even if *TDAR* and *Phinyomark 1* again seemed to be the best sets, i.e., mean accuracy, respectively, of 61.4% and 60.8%, the pair comparison between the sets did not reveal any significant difference (*p* > 0.05), showing a not preferred set when LDA was used as the classifier for this task.

## 4. Discussion

In this study, different intelligent models that combined EMG information extracted from the wrist and forearm were developed and evaluated for solving 30-word hand writing recognition. Although previous studies showed the possibility to transfer myoelectric control scheme in the field of handwriting recognition [10], the analysis was focused on a set of ten digits, whereas in the present work a large dataset containing EMG signals recorded for a large set of words was investigated to better unveil the nature of myoelectric control during handwriting. Indeed, the EMG signals from sensors positioned on the wrist resulted in rich information to characterise complex motor tasks, and they can be combined with standard electrode configurations that limit the acquisition only at the level of the forearm [10,25].

As shown in Figure 2, wrist EMG activity resulted in good quality signals with the amplitude significantly higher in the activation epochs with respect to rest phases. This was encountered in all the subjects analysed, supporting the availability of rich information from data recorded close to the wrist, even if muscle tendon terminations are present in a greater percentage close to the wrist with respect to active fibres [41]. However, the surface EMG probes may also have captured motor commands coming from the active fibres present in connection with the wrist joint [41], eventually providing signals with detectable information [10].

The first pattern recognition test confirmed the hypothesis about the feasibility of using combined forearm and wrist EMG probes for solving handwriting recognition problems with consolidated machine-learning techniques and aggregating state-of-the-art features extracted in TD and FD [1,27,42]. It should be noted that such an experiment confirmed previous results in the literature [9,43], but it also revealed the possibility of using consolidated myoelectric control technology with shallow machine-learning models in the field of handwriting recognition, thus supporting the transfer of methodologies available in the literature for developing human–machine interfaces able to recognize written words by the humans solely based on EMG signals [10,25]. Indeed, the aggregation of a large set of features in the TD and FD domains without a refinement in the processing pipeline (i.e., feature selection, feature reduction, postprocesssing in the classification output) was enough for reaching a classification accuracy greater than 80% for all the subjects in testing for the KNN model (Table 3). Moreover, the trends observed for F1 score and MCC metrics supported the goodness of the accuracy as the principal metric for comparing the results obtained in all the performed experiments since the MCC and F1 showed value that are close to 1, with a trend similar to the accuracy (Table 3).

Another point that should be highlighted in the first pattern recognition experiment is the superior performance of KNN as compared to LDA and SVM, and this appeared consistently among the subjects. This may be imputed to the non-parametric nature of KNN [37], which does not require a priori knowledge of the data distribution and it can fit irregular decision functions due to an increase of complex patterns present in the data. Conversely, both the LDA and linear SVM-based architectures may suffer in fine partitioning the feature space required in this study and are not encountered in typical hand gesture recognition problems where both linear SVM and LDA resulted to be efficient [25,44]. Hence, rather than employ linear approaches, the results suggest the use of nonlinear kernels to better capture the complex hidden pattern within the data. This was confirmed when features were aggregated in specific sets for training and testing the models, as indicated in the second experiment (see Section 2.5). Indeed, the KNN remains the best model with and without the use of the MV postprocessor (Figure 3 and Figure 4), supporting the use of KNN to solve the myoelectric control problem with a large number of classes. However, in the best scenario without MV, KNN reached a mean accuracy lower than 80% when *TDAR* or *Phinyomark 1* was employed (Figure 3), and this may hamper the direct use of the model in a real context. In any case, the investigation of the performances given by specific feature sets was important to better frame the possible line of processing for extracting reliable information from EMG data. Thus, the present study supports *TDAR* as a viable set of EMG characteristics, even when sparse EMG probes are used instead of high-density channels [3]. It is also worth noting that *TDAR* is slightly different from *Hudgins* (see Section 2.3). The main difference lies in the inclusion of VAR WAMP and AR coefficients, which are not present in *Hudgins*. These additional features may be more sensitive to fine motor control commands, which could be more difficult to capture using *Hudgins* [3].

Comparing the KNN accuracy obtained in this work with respect to previous studies in which the letter recognition problem was faced, one can notice that, without the use of MV, the model fed with *TDAR* shows accuracy value greater than the one in [23], where a mean accuracy of 84.3% was obtained through dynamic time warping. However, the model shows lower performance with respect to other approaches proposed in [9,24,28,45], where a mean accuracy greater than 92% was obtained by using techniques based on template matching, modified time warping, and deep-learning approaches. These values of accuracy were reached only when KNN was combined with MV (see Figure 4), where a 99.0% detection accuracy was obtained, making the result comparable with the 98.6% obtained in [28], where auto-encoder for feature extraction and SVM for classification were used with 15 young adults. However, it is important to highlight that in the present study the problem faced has a higher level of complexity. Indeed, the EMG patterns belong to a word in which multiple letters are written consecutively and for which complex motor control planning is actuated by the central nervous system. Moreover, the present classification task took into account 30 classes, i.e., 30 words, which is a high number if compared with the majority of myoelectric pattern recognition studies focused on handwriting recognition, as confirmed in [9]. These considerations not only justify the lower performance obtained in testing (see Figure 3), i.e., not greater than 75%, but also motivates the use of MV for smoothing decision output [27], eventually boosting the performances of the KNN model toward values that can guarantee future validation steps in a real-time context. With all the feature sets, the KNN reached an accuracy value greater than 95%, suggesting the use of the model in practical applications. Furthermore, by comparing the accuracy of KNN in the first and second experiments without MV (respectively, Table 3 and Figure 3), one can recognize that the use of aggregated TD and FD features contributes to the development of classifiers with superior performance compared to the state-of-the-art feature sets employed in the second experiment. This suggests that the use of specific sets, as defined in Section 2.3, can lead to a loss of information that eventually limits the capability of training models to classify unseen data. However, enlarging the number of features increases the complexity of the KNN, indicating the potential use of feature reduction schemes, such as spectral regression methods, when considering a wider range of features, as done in the first experiment [3].

It should be mentioned that the MV technique introduces a time latency of M/2 samples of the decision output since it provides the most probable class knowing M/2 samples before and after the current decision output [27]. Considering the selection of M=4 and considering a feature update rate of 1/0.075=13.33 Hz, this introduces a latency of 150 ms, and it defines a new decision output frequency of 1/0.15=6.66 Hz. Thus, performance improvements were paid at the cost of having a final model with an update frequency that is half of the original one. This aspect may represent a limitation, and it suggests a future research line that can be investigated. Indeed, modern myoelectric interfaces can require output update rates that guarantee efficiency up to 25 Hz. This finds confirmation in [27], where feature update and output decision rates were increased by selecting the *TDAR* feature set together with reduced window length and adopting the high-density EMG sensing technology. A further limitation of the study can be related to the number of subjects recruited to undergo the experiment. However, it should be noted that the number of subjects recruited is coherent with previous studies [9,10]. From this perspective, the enlargement of the dataset may represent a future step toward the generalization of such architecture in real practice, particularly if one is interested in investigating the use of deep-learning techniques [8], which have been shown to be efficient for handwriting recognition applications, even if they were not tested using small EMG window lengths [9]. Moreover, although in this study forearm and wrist EMG channels were used together, future research may focus on comparing the two mentioned locations for automatic handwriting recognition. Indeed, no consensus regarding the optimal location can be found in previous studies [10,25]. While forearm and wrist electrodes provided comparable results in hand gesture recognition problems [25], a drop in performance was observed when transitioning from forearm to wrist in the automatic recognition of handwritten digits [10].

## 5. Conclusions

The study investigated the combination of wrist and forearm EMG data for intelligent handwriting recognition architectures able to decode words from myolelectric signals in healthy young adults. Shallow pattern recognition models, i.e., SVM, LDA, and KNN, were investigated to evaluate the possibility of transfer methodologies typically employed in myeoelctric control in the field of handwriting recognition. Among the aforementioned classifiers, KNN showed the best performances in the pattern recognition experiment proposed, i.e, when a large feature set made of 26 different type of features where considered, and when state-of-the-art feature sets were used with an MV post processor. This research suggests the possibility to develop myoelectric handwriting recognition systems with a consistent number of classes, i.e., 30 words using myoelectric information with approaches that can be used in real scenario. 

## Figures and Tables

**Table 1:** Each subject was asked to handwrite nouns (10), adjectives (10), and verbs (10) according to the experimental protocol described in Section 2.2. To each word corresponds a label number that associates the word with the specific class used in the myoelectric pattern recognition problem.

Nouns		Adjectives		Verbs	
Word	Label	Word	Label	Word	Label
time	1	good	11	be	21
person	2	new	12	have	22
year	3	first	13	do	23
way	4	last	14	say	24
day	5	log	15	get	25
thing	6	great	16	make	26
man	7	little	17	go	27
world	8	own	18	know	28
life	9	other	19	take	29
hand	10	old	20	see	30

**Table 2:** EMG features extracted in the time (16) and frequency (10) domains (TD and FD, respectively), with their abbreviations. More information regarding their computation can be found in [1,10].

Domain	Feature Name	Abbreviation
TD	Integrated EMG	IEMG
	Mean Absolute Value	MAV
	Variance of sEMG	VAR
	Root Mean Square	RMS
	Waveform Length	WL
	Difference Absolute Mean Value	DAMV
	Difference Absolute Standard Deviation Value	DASDV
	Zero Crossing	ZC
	Myopulse Percentage Rate	MYOP
	Willison Amplitude	WAMP
	Slope Sign Change	SSC
	Fuzzy Entropy	FuzEN
	Weighted Permutation Entropy	WPermEN
	Histogram of EMG, 10-bins	HIST
	Auto-Regressive Coefficients, 4th Order	AR
	Cepstrum coefficients of the 4th Order AR process	CC
FD	Mean Frequency	MNF
	Median Frequency	MDF
	Peak Frequency	PKF
	Total Power	TTP
	1st Spectral Moment	SM1
	2nd Spectral Moment	SM2
	3rd Spectral Moment	SM3
	Frequency Ratio	FR
	Power Spectrum Ratio	PSR
	Variance of Central Frequency	VCF

**Table 3:** Acc, F1, and MCC metrics for the first pattern recognition test for all the subjects with KNN, SVM, and LDA classifiers.

Subject	KNN			SVM			LDA		
	Acc	F1	MCC	Acc	F1	MCC	Acc	F1	MCC
1	0.82	0.82	0.82	0.64	0.65	0.62	0.37	0.38	0.32
2	0.87	0.82	0.82	0.66	0.68	0.64	0.33	0.33	0.26
3	0.85	0.85	0.84	0.60	0.61	0.58	0.31	0.31	0.23
4	0.86	0.86	0.85	0.63	0.63	0.61	0.39	0.41	0.35
5	0.83	0.83	0.83	0.63	0.64	0.62	0.38	0.42	0.38
6	0.88	0.88	0.88	0.68	0.68	0.66	0.42	0.42	0.38
Average	0.85	0.84	0.84	0.64	0.65	0.62	0.37	0.37	0.32

## Data Availability

Data are made available upon reasonable request to the authors.
